# Characteristics of mental skills interventions in dance: a mixed methods systematic review protocol

**DOI:** 10.1136/bmjopen-2024-086345

**Published:** 2024-07-29

**Authors:** Michelle Schachtler Dwarika, Mary L Quinton, Sanna Nordin-Bates, Jennifer Cumming

**Affiliations:** 1 School of Sport Exercise and Rehabilitation Sciences, University of Birmingham, Birmingham, UK; 2 Swedish School of Sport and Health Sciences GIH, Stockholm, Sweden

**Keywords:** Systematic Review, MENTAL HEALTH, Protocols & guidelines

## Abstract

**Abstract:**

**Introduction:**

Dancers are expected to navigate major challenges in their careers that might take a toll on their physical and mental health. To address underlying factors that might increase dancers’ mental and physical health difficulties, research suggests the systematic use of techniques to build mental skills that can reduce risk factors and enhance protective factors against the challenges dancers encounter. However, existing mental skills training interventions in dance present a lack of consistency in design, content and duration, making it difficult to provide evidence-based recommendations. Hence, dance researchers and practitioners would benefit from a mixed methods systematic review (MMSR) of the why, what and how of these interventions. Adopting tools such as the Template for Intervention Description and Replication (TIDieR) can aid this endeavour by describing replicable aspects of interventions, thus offering dance researchers suggestions on how to understand, appraise and report intervention characteristics and processes in dance. Therefore, this protocol outlines a MMSR that will employ TIDieR to identify and assess characteristics of mental skills interventions in dance.

**Methods and analysis:**

A systematic search will be undertaken in Psycinfo, Medline, Embase, Sportdiscus, Web of Science and the first 30 pages of GoogleScholar. Following the search, two reviewers will independently screen identified studies in Covidence. One reviewer will extract data using the TIDieR framework and the Mixed Methods Appraisal Tool (MMAT) for quality appraisal, while a second reviewer will check a sample of extracted studies for accuracy. A convergent integrated synthesis will be conducted where quantitative and qualitative evidence will be integrated by qualitising the quantitative data into textual descriptions.

**Ethics and dissemination:**

There is no requirement for ethical approval for this systematic review as no empirical data will be collected. The findings will be disseminated through a peer-reviewed publication in a scientific journal and presentations in several different forums (eg, a dance psychology network, at scientific and applied conferences).

**Prospero registration number:**

CRD42024537249.

**STRENGTHS AND LIMITATIONS OF THIS STUDY**

- This is the first mixed-method systematic review that brings together quantitative and qualitative evidence on mental skills interventions in dance.
- As dance research has been criticised for weak methodological quality in the past, we will offer dance researchers suggestions of how the use of Template for Intervention Description and Replication might be beneficial in planning and reporting more robust dance interventions.
- Due to the focus of the review, we will exclude otherwise interesting insights into mental skills training such as interventions that are dance technique/motor skill focused.
- Non-western dance styles might be under-represented in this review.

## Introduction

 Dancers are expected to meet high physical and mental demands while navigating major challenges in their careers.^1 2^ These can be situational (e.g., major life events, financial concerns), intrapersonal (eg, personality, injury) and interpersonal (e.g., perceptions of educators and audiences) and are often embedded within a sociocultural context that emphasises expectations and pressures to perform and succeed while being subject to critical and perfectionistic attitudes by teachers and performers.^3 4^ Research shows that coping with these challenges is taking a toll on dancers’ physical and mental health and that dancers appear more prone to certain mental health challenges compared with the general population.^5^ For instance, van Winden *et al* indicated that 39.4% of pre-professional contemporary dancers in their study reported mental health issues that affected their mental well-being and physical health.^6^ A more recent study uncovered that 20.8% of the participating professional ballet, contemporary and revue dancers had moderate symptoms of either depression, anxiety or eating disorders and that, compared with a general population, this prevalence was twice as high in female dancers.^5^

To address underlying factors that might increase dancers’ mental and physical health difficulties, research suggests the systematic use of techniques to build mental skills that can reduce risk factors and enhance protective factors against the challenges dancers’ encounter.^7 8^ In sports, mental skills are differentiated from mental qualities, mental techniques and mental skills training (MST).^7 9^ Mental qualities can be viewed as psychological characteristics (e.g., robust confidence, appropriate attentional focus) that facilitate optimal performance and mental well-being.^7 8^ These mental qualities are proposed as the resulting outcomes or the desired mental state achieved through the use of mental skills.^8 10^ Mental skills, on the other hand, are seen as the self-regulatory skills (e.g., maintaining confidence, refocusing, emotional control) that athletes need to manage their cognitive, affective and behavioural state.^8 10^ Mental techniques are methods or procedures (e.g., imagery, self-talk, goal-setting or routines) that athletes engage in to regulate their mental state and develop mental skills.^8 11 12^ Finally, MST (also called psychological skills training) is the learning and implementation of such mental techniques that assist the development of mental skills to achieve performance success and well-being.^7^

Existing evidence in sports and dance indicates that MST might be beneficial for athletes and dancers. For example, in a study involving male youth elite rugby athletes, Sharp *et al.*^10^ found that an MST programme assisted the participants to effectively employ psychological strategies to enhance their performance by increasing the awareness of their behaviours and emotions during rugby games. In dance, Noh *et al*^12^ investigated the effect of an MST intervention on professional ballet dancers’ coping skills and discovered that the combination of imagery, self-talk and relaxation was effective in enhancing coping skills and reducing injury frequency and duration. Moreover, dance literature suggests that other dance professionals, such as educators, found some merit in incorporating MST into their teaching practices.^13 14^ For instance, a study showed that dance educators used mental techniques, like goal setting and imagery, to enhance students’ self-confidence and anxiety regulation and thus encouraged students to actively participate in optimising their performance and well-being.^14^

Despite this compelling evidence, there are no widely recognised manualised MST interventions that might improve dancers’ cognitive, affective and behaviourial states and thus build/maintain beneficial mental qualities for improving mental and physical health. Moreover, existing interventions generally make similar recommendations (e.g., to use a multimodal approach, to offer participants several options for acquiring mental skills) but show significant variations in research design and intervention characteristics.^12 13 15 16^ In terms of research design, one study is solely qualitative^13^ while the others are either mixed-methods^17 18^ or purely quantitative.^19^ In terms of duration and content, one pilot study lasted 6 weeks,^15^ whereas other interventions were conducted over 4 months,^16^ 12 weeks^12^ or even a year.^13^ Two studies involved relevant parties (e.g., dance students and teachers) in their development process,^13 20^ but others relied solely on the sparse, existing literature^15 16^ to shape intervention content. Consequently, researchers might struggle to determine which design, duration and content are appropriate and effective for designing MST interventions in dance.

Due to the seemingly diverse yet valuable evidence, dance researchers and practitioners would benefit from a mixed methods systematic review (MMSR) offering an overview of the why, what and how of effective MST interventions. Understanding what aspects of intervention content and context determine intervention effectiveness will not only provide valuable evidence to inform future intervention design and implementation but also enhance the replicability and sustainability of these interventions.^1 21 22^ Tools, such as the Template for Intervention Description and Replication (TIDieR), can aid this endeavour by describing replicable aspects of interventions, such as who delivers the intervention, how and where the intervention is delivered, whether individual adaptations are used and how well the intervention is adhered to.^23 24^ As such, the TIDieR checklist not only offers authors a structure for describing and reporting interventions but also aids reviewers and editors to assess these descriptions, and makes it easier for readers to use this information.^22 24 25^ Hence, adopting TIDieR in this MMSR can additionally contribute to dance literature by offering dance researchers suggestions how to understand, appraise and report intervention characteristics and processes in dance.^22 24^

## Objectives

The aim of this MMSR is to gain insights into characteristics of existing mental skills interventions in dance and examine this evidence within the TIDieR framework. For this purpose, we aim to answer the following research questions:

1. What are the characteristics of effective mental skills interventions in dance?
2. How does the reporting of these interventions align with the 12 TIDiER items and what, if any, reporting gaps are occurring?
3. What aspects of MST intervention characteristics are associated with improvements in dancers’ mental qualities and physical and mental well-being?

## Methods

This protocol is registered in PROSPERO^26^ and follows elements from the Joanna Briggs Institute’s (JBI) MMSR,^27^ the Preferred Reporting Items for Systematic reviews and Meta-Analyses (PRISMA)^28^ and the Preferred Reporting Items for Systematic reviews and Meta-Analyses Protocols (PRISMA-P) guidance.^29^ The researchers plan to undertake the study from August 2024 to January 2025.

### Eligibility criteria

Studies of interest include quantitative, qualitative or mixed methods approaches. Study designs can include randomised controlled trials (RCTs), non-randomised interventions, quasi-experimental studies, case studies and any qualitative (e.g., case studies, action research, ethnographies) and mixed methods (e.g., convergent, explanatory, sequential, exploratory sequential) research designs.

A preliminary search indicated that without the inclusion of grey literature, the review would not capture the breadth and depth of the information available.^30^ Previous research shows that the inclusion of grey literature will not only minimise publication bias and promote a balanced picture of available evidence but also increase the reviews’ scope and timeliness.^31 32^ Considering these potentially valuable insights and that we are interested in conducting a comprehensive review, this MMSR will include peer-reviewed articles and grey literature with clearly defined method sections, such as book chapters and theses/dissertations. All included studies will be in English, French, German or Scandinavian languages.

The authors of this protocol will follow and use the **P**opulation, **I**ntervention, **C**omparison, **O**utcome principle to inform further eligibility criteria of this review.^27 29^

#### Population

An initial search indicated that including a broad range of individuals engaging in dance at various levels of participation (e.g., pre-professional, professional and recreational) and in different capacities (e.g., dancers and dance educators) would contribute towards more comprehensive insights into MST intervention characteristics in dance. Therefore, this review will include studies that involve dancers and dance professionals (dancers, dance educators) in any dance genre, such as but not limited to, ballet, contemporary, hip hop, modern and jazz dance. Participants included are (a) vocational dancers who dance pre-professionally at vocational dance schools, dance institutions, at the college or university level; (b) professional dancers that either work on a freelance basis or are employed at a professional dance company and are performing regularly to a paying audience; (c) recreational dancers that participate in dance sessions in dance studios or other arenas that offer dance classes; or (d) any type of dance leader (eg, dance educators, artistic directors) who works with any dancers, from recreational to professional level .

Due the authors’ interest in investigating psychological interventions targeting dancers at professional, pre-professional and recreational levels, clinical populations (e.g., individuals that engage in dance for health, rehabilitation or therapy approaches) will be excluded from this review.

#### Intervention

This review will include studies that investigate mental skills interventions in which dancers use mental techniques to acquire or improve mental skills that enhance mental qualities needed to improve performance and mental well-being. To be included, interventions use one or more mental techniques (e.g., imagery, goal setting) or mental skills (e.g., emotion regulation) to enhance mental qualities or mental well-being. Interventions that are dance technique/motor skill focused will be excluded from this review.

#### Comparison

This aspect is not applicable to the current review as no comparison or control groups are being compared.

#### Outcome

Relevant studies will investigate pre- to post-intervention changes in performance (e.g., recovering from injuries), mental health and/or symptoms of common mental disorders (e.g., depression, anxiety) .

‘Mental health’ will be defined according to Keyes’ (2002) dual continua model of mental health, which incorporates the components of emotional well-being, psychological well-being and social well-being. Accordingly, included studies will have measured pre- and post-intervention mental health with scales such as, but not limited to, the Ryff psychological well-being scale and the WHO well-being scale to measure the different dimensions of mental well-being.

Symptoms of common mental disorders in dancers include depression, anxiety and eating disorders and will be measured with scales such as, but not limited to, the Beck Depression Inventory, the Sport Performance Anxiety Scale (SAS-2) or Eating Attitudes Test (EAT-26) pre- and post-intervention.

Potential mechanisms by which the primary outcomes might be achieved include, but are not limited to:

- Changes in mental technique use/ability from pre- to post-intervention and whether these changes are associated with changes in performance, mental health and/or mental illness symptomology.

The review will also identify components of TIDieR that might be helping for explaining changes in primary and secondary outcomes.

### Information sources

The first author will conduct an electronic search of the following databases: Psycinfo (Ovid), Medline (Ovid), Embase (Ovid), SportDiscus (Ebsco) and Web of Science (Clarivate). The searches will take place from inception of the database until August 2024.

### Search strategy

The search strategy has been designed in consultation with a research librarian and will, including all identified keywords, be adapted for each included information source. The search keywords and string are based on a preliminary search and are presented in table 1. Searching will also include (a) the first 30 pages of the search engine GoogleScholar and (b) citation chasing.

**Table 1:** Overview over search strings

Database	Search string
Psycinfo	dance*.mp. OR ballet.mp. or exp Dance/ OR exp Dance/ or college dance.mp. OR exp Dance/ or vocational dance.mp AND exp Skill Learning/ or exp Performance/ or mental skills.mp. AND intervention.mp. or exp Intervention/ OR training.mp. or exp Training/ OR programme.mp.
Medline	dance*.mp OR ballet.mp. or Dancing/ OR Dancing/ or Students/ or collegiate dance.mp. OR Students/ or Dancing/ or vocational dance.mp. AND Athletic Performance/ or mental skills.mp. OR Athletic Performance/ or psychological skills.mp. AND intervention.mp. OR training.mp. OR programme.mp.
Embase	dance*.mp.OR dancing/ or vocational dance.mp OR ballet dancer/ or ballet.mp. or dancing/OR collegiate dancer.mp. AND mental skills.mp. or mental performance/ or skill/ OR skill/ or psychological skills.mp. AND Intervention.mp. or intervention study/ or psychosocial intervention/ OR programme development/ or Programme.mp. OR training/ or Training.mp.
Sportdiscus	AB dance* AND TI programme OR TI training AND mental skills OR psychological skills AND AB intervention
Web of science	dance* (Topic) and **Dance** (Should – Search within topic) and **Ballet** (Should – Search within topic) and **Dancing** (Should – Search within topic) and **Contemporary Dance** (Should – Search within topic) and **Dancers** (Should – Search within topic) and **Dance Education** (Should – Search within topic) AND mental skills* (Topic) and **Mental Skills** (Should – Search within topic) and **Coping Skills** (Should – Search within topic) and **Mental Training** (Should – Search within topic) and **Mental Skills Training** (Should – Search within topic) and **Psychological Skills** (Should – Search within topic)OR psychological skills* (Topic) and **Psychological Skills** (Should – Search within topic) and **Coping Skills** (Should – Search within topic) and **Mental Skills** (Should – Search within topic) and **Psychological Skills Training** (Should – Search within topic) and **Psychological Performance** (Should – Search within topic) AND **intervention** (Topic) and **Intervention** (Should – Search within topic) and **Interventions** (Should – Search within topic) OR **training** (Topic) and **Training** (Search within topic) and **Education** (Should – Search within topic) OR programme (Topic)
Google scholar	psychological skills OR mental skills AND interventions OR ballet

### Data management

Covidence, a web-based systematic review platform developed to guide reviewers through the systematic review workflow, will be used to screen, assess and extract the studies.^33^ Extraction and assessment tables and a PRISMA flow diagram will be created in Covidence and adapted accordingly.^33^

### Selection process

Following the search, all identified studies will be loaded into Covidence and duplicates removed. Titles and abstracts will then be screened by the first and fourth authors for the assessment against the inclusion criteria for the review. The full text of selected citations will be assessed in detail against the inclusion criteria by the first and fourth author. Based on the inclusion/exclusion criteria, studies will be sorted into ‘inclusion’, ‘exclusion’, ‘full-text review’ or ‘irrelevant’.

Reasons for exclusion of full-text studies that do not meet the inclusion criteria will be recorded and reported in the systematic review. Any disagreements that arise between the reviewers at each stage of the study selection process will be resolved through discussion or with the second author. The results of the search will be reported in full in the final review and presented in a PRISMA flow diagram.^28^

### Data extraction

The first author will use the TIDieR framework and its 12 checklist headings (see online supplemental table 2) to extract data from studies included in the review.^24^ The use of the TIDieR headings will allow us to document disparities and similarities of the studies’ documented intervention characteristics.^34^ Additionally, the first author will extract characteristics of participants involved in each study and the outcomes (see table 2) to determine if the MST intervention was effective. The second author will check 30% of the extracted studies for accuracy.

**Table 2:** Items used to extract data on intervention effectiveness

Reference	Full change (n=). All of the dependent variables (DVs) are positively and significantly changed from pre- to post-intervention, and all changes are significantly greater than any control conditions.	Moderate change (n=). One or more DVs positively and significantly changed from pre- to post-intervention (significantly greater than any control conditions), and one or more DVs did not change, changed negatively or did not differ from a control, from pre- to post-intervention.	No change (n=). None of the DVs positively or significantly changed from pre- to post-intervention or changes are not significantly greater than any control conditions.

Any disagreements that arise between the reviewers will be resolved through discussion or with the fourth author. Authors of papers will be contacted to request missing or additional data, where required.

### Risk-of-bias assessment

The quality of all included studies, including those from the grey literature, will be appraised using the Mixed Methods Appraisal Tool (MMAT).^35^ The MMAT is a versatile appraisal tool that considers all necessary elements for different data sources and evaluates studies in five categories: qualitative research, RCTs, non-randomised studies, quantitative descriptive studies and mixed methods studies (see online supplemental table 4).

The first and second authors will independently judge each study, including verbatim quotes, according to each domain in the tool. Judgements as to the possible risk of bias will be rated as ‘yes’ or ‘no’. If insufficient detail is provided, the risk will be judged as ‘can’t tell’. If required, authors of papers will be contacted (on two separate occasions, 2 weeks apart via e-mail) to request missing or additional data for clarification. Any disagreements that arise between the reviewers will be resolved through discussion or with the fourth author. The results of critical appraisal will be reported in narrative form and in a table.

All studies, regardless of the results of their methodological quality, will undergo data extraction and synthesis. The results of the critical appraisal will be incorporated into the certainty assessment to detail the strength of findings associated with each outcome of interest.

### Data synthesis and integration

This review will follow a convergent integrated approach according to the JBI methodology for MMSR and apply these three steps^27^ :

In step one, the reviewers will consider the final phases of both the quantitative and qualitative evidence and examine if the findings naturally complement each other. Then, quantitative data will be transformed into textual descriptions of study and intervention characteristics (qualitised) and combined with qualitative findings using the same categorisations.^36^

In step two, the assembled data will be pooled and structured according to the 12 TIDieR checklist items. Both the qualitative and qualitised data will provide evidence and an overview of intervention characteristics that have been reported or overlooked.

In step three, the reviewers will produce a preliminary narrative synthesis of the results. Findings will be structured and reported according to the 12 TIDieR checklist items. Data on intervention characteristics associated with improvements in dancers’ mental qualities and physical and mental health will be synthesised and reported separately.

### Confidence in cumulative evidence

Levels of evidence and Confidence in the Evidence from Reviews of Qualitative Research will be used to assess the strength of evidence of each study and integrated into the table assessing the studies’ methodological quality.^37 38^

### Patient and public involvement

None

## supplementary material

10.1136/bmjopen-2024-086345 online supplemental file 1

10.1136/bmjopen-2024-086345 online supplemental file 2
