# Supplementary material for: Characteristics of mental skills interventions in dance: a mixed methods systematic review protocol
Source: BMJ Open. 2024 Jul 29;14(7):e086345. doi: 10.1136/bmjopen-2024-086345 (PMC11288147; doi:10.1136/bmjopen-2024-086345)
Supplement: online supplemental file 1 [file bmjopen-14-7-s001.pdf]

Table 2: TIDieR items and headings used to extract the data

**Reference**	***Brief Name*** (or phrase describing the intervention)	***Why*** (rationale, theory, or goal essential to intervention)	***What materials*** (physical or informational materials used, and where they can be accessed)	***What procedure*** (activities, or processes used in the intervention)	***Who*** (background, expertise of provider, and training given)	***How*** (modes of delivery to group or individual)	***Where*** *(*type of location)	***When and How Much*** (number of times, sessions, intensity and over what time period delivered)	***Tailoring*** (what, why, when, and how of planned personalisation/adaptation)	***Modification*** what, why, when and how of intervention modification during study)	***How well planned*** (if intervention adherence or fidelity was assessed, describe how and by whom)	***How well actual*** (describe the extent to which the intervention was delivered as planned)
