# Supplementary material for: Characteristics of mental skills interventions in dance: a mixed methods systematic review protocol
Source: BMJ Open. 2024 Jul 29;14(7):e086345. doi: 10.1136/bmjopen-2024-086345 (PMC11288147; doi:10.1136/bmjopen-2024-086345)
Supplement: online supplemental file 2 [file bmjopen-14-7-s002.pdf]

Study	Title	All types of studies: Are there clear research questions?	All types of studies: Do the collected data allow to address the research questions?	Qualitative 1: Is the qualitative approach appropriate to answer the research question?	Qualitative 2: Are the qualitative data collection methods adequate to address the research question?	Qualitative 3: Are the findings adequately derived from the data?	Qualitative 4: Is the interpretation of results sufficiently substantiated by data?	Qualitative 5: Is there coherence between qualitative data sources, collection, analysis and interpretation ?	Quantitative non randomized 1: Are the participants representative of the target population?	Quantitative non randomized 2: Are measurements appropriate regarding both the outcome and intervention (or exposure)?	Quantitative non randomized 3: Are there complete outcome data?	Quantitative non randomized 4: Are the confounders accounted for in the design and analysis?	Quantitative non randomized 5: During the study period, is the intervention administered (or exposure occurred) as intended?	Quantitatie randomized 1: Is randomization appropriately performed?	Quantitatie randomized 2: Are the groups comparable at baseline?	Quantitatie randomized 3: Are there complete outcome data?	Quantitatie randomized 4: Are outcome assessors blinded to the intervention provided?	Quantitatie randomized 5: I Did the participants adhere to the assigned intervention?	Quality assessment (LoE and CerQual)
	.																		
**Summary**		Clear research question: N=<br>Yes (n=)<br>No (n=)<br>Reasons:<br>(n=)<br><br>Data that answers question<br>Yes (n =)<br>No (n=)<br>Reason 1:		Qualitative summary:<br>N =<br><br>Between study analysis						Quantitative non random<br>N=<br><br>Between study analysis				Quantitative RCT<br>N=					
