# Supplementary material for: Characteristics of mental skills interventions in dance: a mixed methods systematic review protocol
Source: BMJ Open. 2024 Jul 29;14(7):e086345. doi: 10.1136/bmjopen-2024-086345 (PMC11288147; doi:10.1136/bmjopen-2024-086345)
Supplement: Supplementary file 3 [file bmjopen-2024-086345.reviewer_comments.pdf]

PEER REVIEW HISTORY

BMJ Open publishes all reviews undertaken for accepted manuscripts. Reviewers are asked to complete a checklist review form (**http://bmjopen.bmj.com/site/about/resources/checklist.pdf**) and are provided with free text boxes to elaborate on their assessment. These free text comments are reproduced below.

ARTICLE DETAILS

**TITLE (PROVISIONAL)**	Characteristics of mental skills interventions in dance: a mixed methods systematic review protocol
**AUTHORS**	Dwarika, Michelle; Quinton, Mary; Nordin-Bates, Sanna; Cumming, Jennifer

VERSION 1 – REVIEW

**REVIEWER**	Birtwell, Kelly<br>Manchester University, Primary Care Research
**REVIEW RETURNED**	26-Apr-2024

**GENERAL COMMENTS**	This manuscript reports a protocol for a mixed methods systematic review of mental skills interventions for dancers. This is an interesting topic and the review has potential to make a useful contribution to the field. Some sections of the manuscript are well written whereas other areas require clarification. There are some minor typographical errors throughout, e.g. the first letters of some sentences are not capitalised, and there is a random question mark in the middle of one sentence. I offer the following comments to enhance the overall clarity and value to the readership.<br><br>Introduction:<br>- P.8, paragraph 2: this could benefit from rephrasing to improve the clarity. E.g. this paragraph says there are 'no established MST interventions' yet the previous paragraph refers to some interventions, and this paragraph refers to others. Is the intended meaning that there is significant variation and no widely recognised manualised MST interventions, or no research studies on MST interventions, or no evidence for the effectiveness of particular MST interventions? The paragraph goes on to say a scoping review 'supports this gap'. Does this mean the scoping review confirms there is a gap, or it provides evidence to address that gap?<br>- P.8 line 30 and 41: I suggest using 'stakeholders' rather than 'shareholders'.<br>- P.8 line 34-49: when discussing 'designs, duration and content' please distinguish between intervention characteristics and study design characteristics. These are currently conflated yet are two different things.<br>- P.8 45: "Consequently, it is hard to disseminate the appropriate design,…" the word 'disseminate' does not make sense in the context of this sentence.<br><br>Methods:<br>- The methods state a range of grey literature will be included. However, it is not clear how the inclusion of this literature will help to answer the research questions, how this literature will be

	assessed for quality, and how data from this literature will be incorporated into the review.<br>- The introduction focuses on MST for dancers who dance for their career. However the methods section states studies on recreational dancers, recreational dance leaders, and artistic directors will be included. The rationale for this is not clear.<br>- I suggest changing 'data collection' to 'data extraction'.<br>- Please clarify the percentage of extracted studies the second author will check.<br>- It is not clear how the authors plan to answer research question 3, about intervention characteristics associated with improvements in dancers' health and wellbeing. Will this be according to findings reported in the included papers, or will the authors perform any analyses to determine this? Please provide further information.

**REVIEWER**	Benoît-Piau, Justine<br>Amsterdam UMC Locatie VUmc
**REVIEW RETURNED**	24-May-2024

**GENERAL COMMENTS**	Characteristics of mental skills interventions in dance: a mixed methods systematic review protocol<br><br>General<br><br>This is a protocol for a mixed methods systematic review protocol. The aim is to gain insight into characteristics of existing mental skills interventions in dance and examine this evidence within the TIDieR framework.<br><br>Novelty: From a Prospero, Google Scholar and PubMed search, this seems to add to existing literature as there seems to be no SR/MAs on that subject. However, I am unsure there are enough studies on the subject to do a systematic review.<br><br>Significance: Authors highlight that they will systematically review mental skills interventions using TIDieR to assess different aspects of the interventions to inform future intervention design and implementation. Indeed, multiple dance studies do not use robust methodologies and this review could provide valuable insights for future research. Maybe less significant for clinicians and/or dance educators.<br><br>All tables in the main manuscript are not referenced in the text (for example, instead of "Based on a preliminary search, the search keywords and string will look as follows", please use "The search keywords and string are based on a preliminary search and are presented in Table 1."). They should be.<br><br>Abstract<br><br>Please review instructions to authors: abstract should be unstructured, 250 words maximum, and the key messages required for protocol are not present.<br><br>Line 16-17: remove the apostrophe after dancers.<br><br>Embase is mentioned in methods here, but not in methods in the main manuscript.

	You state that quantitative and qualitative evidence will be analyzed separately first. Yet, in the data synthesis section of Methods, you only describe the integration of qualitized quantitative data.<br><br>Are other dissemination strategies planned? Knowledge transfer to dance professionals, dancers, conference abstracts?<br><br>The second limitation is missing a period at the end of the sentence.<br><br><br><br>Introduction<br><br>I am unsure why the introduction is structured in rationale and objectives. It seems unnecessary.<br><br>In this context, “dance educator” seems a preferable term to “teacher”.<br><br>“Maybe unsurprisingly,” seems superfluous.<br><br>For the two last sentences of the first paragraph, please clarify which dancer population you are talking about (professional, preprofessional, ballet, contemporary, hip hop…). Does this prevalence vary widely from general population?<br><br>Last sentence of first paragraph should start with a capital letter.<br><br>First sentence of second paragraph seems to be word for word what is stated in the abstract. Please rephrase one or the other.<br><br>Please add references to first sentence of this paragraph.<br><br>Second sentence of third paragraph: Please mention if these are mental challenges (I am assuming yes).<br><br>Third sentence of third paragraph: what dance style?<br><br>Line 34-35 fourth paragraph: Please add a capital letter.<br><br>First paragraph of page 5, lines 11-16: There seems to be a random “?” in the middle of the sentence.<br><br>Specific: Two references in the introduction seem to not be well formatted (last paragraph of page 7 and first paragraph of page 8).<br><br>Objectives<br><br>Specific: Please add capital T to “The aim…” in objectives.<br><br>Having read the methods, I am unsure how you measure the “extent” of how the interventions align with TIDieR items.<br><br>I am also failing to see in the methods how you will answer this 3rd objective.

	Methods<br><br>Page 9 lines 46-48: Will be registered in PROSPERO, although I am not sure why it is not already registered. Does not mention that will follow PRISMA guidelines. Also, they mention they follow element from the Joanna Briggs Institute’s MMSR and PRISMA-P guidance but have not attached the files to detail which elements are followed and why (i.e. some might be not applicable). Also, the PRISMA-P abbreviation should be defined before use.<br><br>Eligibility criteria<br><br>Please change “randomized control trials” to “randomized controlled trials”.<br><br>Also, the second sentence seems to be referring only to quantitative methodologies. What kind of qualitative and/or mixed methods study design are going to be included?<br><br>In the second paragraph, why include grey literature if it is stated as limitations? Are there not enough studies in the literature?<br><br>Eligibility criteria – population<br><br>First sentence: I would prefer “dance educator” instead of “teacher”.<br><br>Could you add a sentence on why you include such a broad population?<br><br>Could you briefly justify your exclusion criteria?<br><br>Eligibility criteria – intervention & outcome<br><br>In intervention, you state that interventions that are dance technique/motor skill focused will be excluded. In outcomes you state that relevant studies will seek to improve dancer’s physical health. Is this because some mental skills/techniques could have physical outcomes? If yes, please make it clear in intervention or outcome.<br><br>Information sources<br><br>Please update the end date with the acceptance of this protocol.<br><br>Search strategy<br><br>What search strategy will you follow in Google Scholar?<br><br>Selection process<br><br>Please define PRISMA in the Data management paragraph and use only abbreviation thereafter.<br><br>Data collection process<br><br>Second line, the reference does not seem to be well formatted.<br><br>Table 2: Will you include n?

	What is Table 3 based on? Why is it not referenced in the text? I don't understand Table 3…<br><br>Risk of bias assessment<br><br>I do not see any Appendix. Also, is there a table 1 in Appendix? There is no reference to it in the text.<br><br>The title of Table 4 is not with the actual Table.<br><br>Data synthesis and integration<br><br>Please detail the process to transform quantitative data into textual description.<br><br>Similarly, how do you "assemble" the qualitized data with the qualitative data?<br><br>Confidence in cumulative evidence<br><br>Please mention which table you are talking about.<br><br>**Please add a section for ethical considerations before the discussion.<br><br>Discussion<br><br>Please explain why inclusion of grey literature and excluding dance techniques/ motor skill interventions are limitations and why you choose to still uphold these criteria/methods.<br><br>This could be helpful: https://doi.org/10.1136/bmjsem-2023-001784.<br><br>Please reference your first sentence.<br><br>Please reference the sentence "Also, based on the scope of existing evidence, …".<br><br>References<br><br>Some references seem to be incomplete (3, 12, 21, 31, 37…).<br><br>Thank you for the opportunity to review this manuscript.

VERSION 1 – AUTHOR RESPONSE

Reviewer: 1
Dr. Kelly Birtwell, Manchester University
Comments to the Author:

This manuscript reports a protocol for a mixed methods systematic review of mental skills interventions for dancers. This is an interesting topic and the review has potential to make a useful contribution to the field. Some sections of the manuscript are well written whereas other areas require clarification. There are some minor typographical errors throughout, e.g.

We are happy to hear that there is interest in this topic and that the review could be a useful contribution to the field.

the first letters of some sentences are not capitalised, and there is a random question mark in the middle of one sentence.

Thank you for making us aware of this. The manuscript has now been proof-read and mistakes have been amended.

I offer the following comments to enhance the overall clarity and value to the readership.

Thank you for your very helpful and engaging comments! We think the manuscript has greatly improved as a result of your comments.

Introduction:
1. P.8, paragraph 2: this could benefit from rephrasing to improve the clarity. E.g. this paragraph says there are ‘no established MST interventions’ yet the previous paragraph refers to some interventions, and this paragraph refers to others. Is the intended meaning that there is significant variation and no widely recognised manualised MST interventions, or no research studies on MST interventions, or no evidence for the effectiveness of particular MST interventions?

Thank you so much for this interesting and valuable comment. We agree that rephrasing this part of the manuscript will improve clarity! Your suggestions have now been incorporated into the paragraph, and it reads as follows:
“Despite this compelling evidence, there are no widely recognised manualised MST interventions that might improve dancers’ cognitive, affective and behavioural states and thus build/maintain beneficial mental qualities for improving mental and physical health. Moreover, existing interventions generally make similar recommendations (e.g., to use a multimodal approach, to offer participants several options for acquiring mental skills) but show significant variations in research design and intervention characteristics(1, 16, 18-20). In terms of research design, one study is solely qualitative (1) while the others are either mixed-methods (21, 22) or purely quantitative (23). In terms of duration and content, one pilot study lasted six weeks (18), whereas other interventions were conducted over four months (19), twelve weeks (16), or even a year (1). Two studies involved involved relevant parties (e.g., dance students and teachers) in their development process (1, 20), but others relied solely on the sparse, existing literature (18, 19) to shape intervention content. Consequently, researchers might struggle to determine which design, duration and content are appropriate and effective for designing MST interventions in dance.“
2. The paragraph goes on to say a scoping review ‘supports this gap’. Does this mean the scoping review confirms there is a gap, or it provides evidence to address that gap?

Thank you for making us aware of this. To improve the clarity of the paragraph, we have removed that particular text.

3. P.8 line 30 and 41: I suggest using ‘stakeholders’ rather than ‘shareholders’.

. Thank you for this suggestion. Stakeholders is seen as a controversial term which we would like to substitute with a more agreeable and appropriate description of partnership. This is not only to

address our positionality as white females, but also to acknowledge that the term stakeholders might trigger unwanted associations of colonialism and injustice.

We have based this decision on these explanations:

https://www.linkedin.com/pulse/time-adopt-new-word-stakeholders-marie-gettel-gilmartin/

https://www.fasttrackimpact.com/post/why-we-shouldn-t-banish-the-word-stakeholde

As a result, we have opted to substitute shareholders/stakeholders with “involved relevant parties”.

4. P.8 line 34-49: when discussing ‘designs, duration and content’ please distinguish between intervention characteristics and study design characteristics. These are currently conflated yet are two different things.

Thank you for this very valuable comment. We have now rephrased this into: “…but others relied solely on the sparse, existing literature (18, 19) to shape intervention content.”.

5. P.8 45: “Consequently, it is hard to disseminate the appropriate design,…” the word ‘disseminate’ does not make sense in the context of this sentence.

Thank you for this comment. We have amended this sentence to: “Consequently, researchers might struggle to determine which design, duration and content are appropriate and effective for designing MST interventions in dance.“

Methods:
6. The methods state a range of grey literature will be included. However, it is not clear how the inclusion of this literature will help to answer the research questions, how this literature will be assessed for quality, and how data from this literature will be incorporated into the review.

These are a very essential and interesting points that we have tried to elaborate on in different parts of the revised manuscript.

We now more clearly explain that grey literature will be included in the systematic review to ensure that it covers the breadth of available MST interventions. Data from all included studies (whether this is grey or peer-reviewed) will be pooled and analysed according to the JBI MMSR guidelines. The MMAT will be applied to all forms of included literature to evaluate its quality. This is a versatile tool that is compatible with a MMSR and has previously been used to evaluate grey literature such as in this example below:

Wanless, B., Berry, A., & Noblet, T. (2022). Self-management of musculoskeletal (MSK) conditions: What is most useful to patients? Protocol for a mixed methods systematic review. Musculoskeletal Care, 20(2), 271-278.

By including the grey literature, we will capture potential differences in grey and peer reviewed literature.

In the manuscript, we now explain the inclusion of grey literature in more detail and how doing so will contribute to the review/answering the research questions as follows:

“A preliminary search indicated that without the inclusion of grey literature, the review would not capture the breadth and depth of the information available (33). Previous research shows that the

inclusion of grey literature will not only minimize publication bias and promote a balanced picture of available evidence, but also increase the reviews' scope and timeliness (34, 35). Considering these potentially valuable insights and that we are interested in conducting a comprehensive review, this MMSR will include peer-reviewed articles and grey literature with clearly defined method sections, such as book chapters and theses/dissertations. All included studies will be in English, French, German, or Scandinavian languages."

Addressing how the grey literature will be assessed now reads as follows:

"The quality of all included studies, including those from the grey literature, will be appraised using the Mixed Methods Appraisal Tool (MMAT)(38). The MMAT is a versatile appraisal tool that considers all necessary elements for different data sources and evaluates studies in five categories: qualitative research, randomized controlled trials, non-randomized studies, quantitative descriptive studies, and mixed methods studies (see Table 4). "

7. The introduction focuses on MST for dancers who dance for their career. However the methods section states studies on recreational dancers, recreational dance leaders, and artistic directors will be included. The rationale for this is not clear.

Thank you, this was a valuable point which we have tried to address and clarify in the amended manuscript, which now reads as follows:

"An initial search indicated that including a broad range of individuals engaging in dance at various levels of participation (e.g., pre-professional, professional and recreational) and in different capacities (e.g., dancers and dance educators) would contribute towards more comprehensive insights into MST intervention characteristics in dance. Therefore, this review will include studies that involve dancers and dance professionals (dancers, dance educators) in any dance genre, such as but not limited to, ballet, contemporary, hip hop, modern and jazz dance. Participants included are: (a) vocational dancers who dance pre-professionally at vocational dance schools, dance institutions, at college or university level; (b) professional dancers that either work on a freelance basis or are employed at a professional dance company and are performing regularly to a paying audience; (c) recreational dancers that partake in dance sessions in dance studios or other arenas that offer dance classes; or (d) any type of dance leader (e.g., dance educators, artistic directors) who works with any dancers, from recreational to professional level ."

8. I suggest changing 'data collection' to 'data extraction'.

Amended in the revised manuscript

9. Please clarify the percentage of extracted studies the second author will check.

Amended. This now reads:
"The second author will check 30% of the extracted studies for accuracy."
10. It is not clear how the authors plan to answer research question 3, about intervention characteristics associated with improvements in dancers' health and wellbeing. Will this be according to findings reported in the included papers, or will the authors perform any analyses to determine this? Please provide further information.

Thank you for this valuable input. We have tried to clarify this point in the outcome section of the manuscript, which now reads as follows:

“Relevant studies will investigate pre- to post intervention changes in performance (e.g. recovering from injuries), mental health, and/or symptoms of common mental disorders (e.g., depression, anxiety).

"Mental health" will be defined according to Keyes' (2002) dual continua model of mental health, which incorporates of the components of emotional well-being, psychological well-being, and social well-being. Accordingly, included studies will have measured pre- and post-intervention mental health with scales such as, but not limited to, the Ryff psychological well-being scale and the WHO wellbeing scale to measure the different dimensions of mental well-being.

Symptoms of common mental disorders in dancers include depression, anxiety, and eating disorders and will be measured with scales such as, but not limited to, the Beck Depression Inventory, the Sport Performance Anxiety Scale (SAS-2) or Eating Attitudes Test (EAT-26) pre-and post-intervention.

Potential mechanisms by which the primary outcomes might be achieved include, but not limited to:

- changes in mental technique use/ability from pre- to post-intervention and whether these changes are associated with changes in performance, mental health, and/or mental illness symptomology

The review will be also identify components of TIDieR that might be helping for explaining changes in primary and secondary outcomes.

Reviewer: 2
Miss Justine Benoît-Piau, Amsterdam UMC Locatie VUmc
Comments to the Author:

General
This is a protocol for a mixed methods systematic review protocol. The aim is to gain insight into characteristics of existing mental skills interventions in dance and examine this evidence within the TIDieR framework.

Novelty: From a Prospero, Google Scholar and PubMed search, this seems to add to existing literature as there seems to be no SR/MAs on that subject. However, I am unsure there are enough studies on the subject to do a systematic review.

Significance: Authors highlight that they will systematically review mental skills interventions using TIDieR to assess different aspects of the interventions to inform future intervention design and implementation. Indeed, multiple dance studies do not use robust methodologies and this review could provide valuable insights for future research. Maybe less significant for clinicians and/or dance educators.

We are happy to hear that you also think this review could provide valuable insights for future research!

All tables in the main manuscript are not referenced in the text (for example, instead of “Based on a preliminary search, the search keywords and string will look as follows”, please use “The search keywords and string are based on a preliminary search and are presented in Table 1.”). They should be.

Thank you for this valuable input. We have amended the manuscript throughout and have now referenced all tables in the text.

Abstract
11. Please review instructions to authors: abstract should be unstructured, 250 words maximum, and the key messages required for protocol are not present.

Thank you for making us aware of this. We have worked according to the following instructions and in accordance with the editor's wishes: https://bmjopen.bmj.com/pages/authors#protocol

These instructions state: "Abstract: this should be structured with the following sections. Introduction; Methods and analysis; Ethics and dissemination. Registration details should be included as a final section, if appropriate".

However, we agree with you that the PROSPERO registration is missing and have amended this point in the manuscript.

12. Line 16-17: remove the apostrophe after dancers.

This has been amended.

13. Embase is mentioned in methods here, but not in methods in the main manuscript.

This has been amended and is highlighted in the revised manuscript.

14. You state that quantitative and qualitative evidence will be analyzed separately first. Yet, in the data synthesis section of Methods, you only describe the integration of qualitized quantitative data.

Thank you for this valuable input. We have addressed this section as follows:

In the abstract, the relevant sentence now reads:

"A convergent integrated synthesis will be conducted where quantitative and qualitative evidence will be integrated by qualitizing the quantitative data into textual descriptions."
In the synthesis section now reads:
"This review will follow a convergent integrated approach according to the JBI methodology for MMSR and apply these three steps (31):
In step one, the reviewers will consider the final phases of both the quantitative and qualitative evidence and examine if findings naturally complement each other. Then, quantitative data will be transformed into textual descriptions of study and intervention characteristics (qualitized) and combined with qualitative findings using the same categorisations (37).»
15. Are other dissemination strategies planned? Knowledge transfer to dance professionals, dancers, conference abstracts?

Thank you for this input. We have added to the existing information and the manuscript now reads:

"The findings will be disseminated through a peer-reviewed publication in a scientific journal and presentations in several different forums (e.g., a dance psychology network, at scientific and applied conferences)."
16. The second limitation is missing a period at the end of the sentence.

This has been amended in the manuscript.

Introduction

17. I am unsure why the introduction is structured in rationale and objectives. It seems unnecessary.

Thank you for your valuable observation and comment. We have structured the whole protocol according to PRISMA guidelines: https://www.equator-network.org/reporting-guidelines/prisma/

These guidelines recommend structuring the introduction with “rationale” and “objectives”. Yet, we see that the “rationale” bit might seem superfluous and therefore suggest removing “rationale” but to keep “objectives”. This will aid the reader to interpret the information given in the introduction.

18. In this context, “dance educator” seems a preferable term to “teacher”.

Thank you for this input. This has been amended in the manuscript.

19. “Maybe unsurprisingly,” seems superfluous.

This has been amended in the manuscript.

20. For the two last sentences of the first paragraph, please clarify which dancer population you are talking about (professional, preprofessional, ballet, contemporary, hip hop…). Does this prevalence vary widely from general population?

Thank you for this interesting question. There are only a few studies comparing mental health or illness between dancers and non-dancers. Therefore, we didn’t want to speculate but tried to show the importance to be aware of and address these issues. The study cited regarding this point concluded that female dancers’ prevalence for mental health challenges were twice as high compared to a general population. To address this and the other valuable points, we have amended the manuscript as follows:
“For instance, van Winden et al. indicated that 39.4% of pre-professional contemporary dancers in their study reported mental health issues that affected their mental well-being and physical health (10). A more recent study uncovered that 20.8% of the participating professional ballet, contemporary and revue dancers had moderate symptoms of either depression, anxiety or eating disorders and that, compared to a general population, this prevalence was twice as high in female dancers (9).”
21. Last sentence of first paragraph should start with a capital letter.

Thank you. This was amended in the revised manuscript.

22. First sentence of second paragraph seems to be word for word what is stated in the abstract. Please rephrase one or the other.

Amended in the abstract. The sentence now reads as follows:

“To address underlying factors that might increase dancers’ mental and physical health difficulties, research suggests the systematic use of techniques to build mental skills that can reduce risk factors and enhance protective factors against the challenges dancers’ encounter.”

23. Please add references to first sentence of this paragraph.

References are now included in this first sentence.

24. Second sentence of third paragraph: Please mention if these are mental

challenges (I am assuming yes).

Thank you for this valuable pointer. To ensure clarity, this has been amended to:

“For example, in a study involving male youth elite rugby athletes, Sharp et al. (2013) found that an MST program assisted the participants to effectively employ psychological strategies to enhance their performance by increasing awareness of their behaviours and emotions during rugby games.”

25. Third sentence of third paragraph: what dance style?

This has been amended in the revised manuscript.

26. Line 34-35 fourth paragraph: Please add a capital letter

Thank you. This paragraph has been amended has requested by reviewer 1.

27. First paragraph of page 5, lines 11-16: There seems to be a random “?” in the middle of the sentence.

Thank you. This mistake has been amended.

28. Specific: Two references in the introduction seem to not be well formatted (last paragraph of page 7 and first paragraph of page 8).

Thank you for pointing this out to us. Several references have now been amended and should be now formatted correctly.

Objectives
29. Specific: Please add capital T to “The aim…” in objectives.

Amended in the revised manuscript.

30. Having read the methods, I am unsure how you measure the “extent” of how the interventions align with TIDieR items.

Agreed, “the extent” might be a confusing description to use. Therefore, we have addressed this input by rephrasing the question to:

“How does the reporting of these interventions align with the 12 TIDiER items and what, if any, reporting gaps are occurring?”

31. I am also failing to see in the methods how you will answer this 3rd objective.

Please see our response to Comment #10 from Reviewer 1

Methods

32. Page 9 lines 46-48: Will be registered in PROSPERO, although I am not sure why it is not already registered.

Thank you for this question. The PROSPERO registration was slightly delayed but has been completed during the reviewing process of this protocol. It is now mentioned and referenced in the revised manuscript.

33. Does not mention that will follow PRISMA guidelines. Also, they mention they follow element from the Joanna Briggs Institute's MMSR
and PRISMA-P guidance but have not attached the files to detail which elements
are followed and why (i.e. some might be not applicable). Also, the PRISMA-P
abbreviation should be defined before use

This has been addressed and amended as follows:

…"and follows elements from the Joanna Briggs Institute's (JBI) MMSR (30), the Preferred Reporting Items for Systematic reviews and Meta-Analyses (PRISMA) (31) and the Preferred Reporting Items for Systematic reviews and Meta-Analyses Protocols (PRISMA-P) guidance"

The PRISMA-P checklist has been added to this revision

Eligibility criteria
34. Please change "randomized control trials" to "randomized controlled trials".

Amended

35. Also, the second sentence seems to be referring only to quantitative
methodologies. What kind of qualitative and/or mixed methods study design are
going to be included?

Thank you. We have amended the text to:

…"and any qualitative (e.g., case studies, action research, ethnographies) and mixed methods (e.g., convergent, explanatory, sequential, exploratory sequential) research designs."

36. In the second paragraph, why include grey literature if it is stated as limitations?
Are there not enough studies in the literature?

This a very good point that we have tried to elaborate on in the revised manuscript. Please see our response to Comment #6 from Reviewer 1.

As this can also been as a strength (and limitation) we have removed the grey literature point from "limitations" in the abstract.

Eligibility criteria – population
37. First sentence: I would prefer "dance educator" instead of "teacher".

Amended.

38. Could you add a sentence on why you include such a broad population?

This was a very valuable point that we have addressed in the amended manuscript as follows:

"An initial search indicated that including a broad range of individuals engaging in dance at various levels of participation (e.g., pre-professional, professional and recreational) and in different capacities

(e.g., dancers and dance educators) would contribute towards more comprehensive insights into MST intervention characteristics in dance. Therefore, this review will include studies that involve dancers and dance professionals (dancers, dance educators) in any dance genre, such as but not limited to, ballet, contemporary, hip hop, modern and jazz dance. Participants included are: (a) vocational dancers who dance pre-professionally at vocational dance schools, dance institutions, at college or university level; (b) professional dancers that either work on a freelance basis or are employed at a professional dance company and are performing regularly to a paying audience; (c) recreational dancers that partake in dance sessions in dance studios or other arenas that offer dance classes; or (d) any type of dance leader (e.g., dance educators, artistic directors) who works with any dancers, from recreational to professional level ."

39. Could you briefly justify your exclusion criteria?

We now briefly justify the exclusion criteria using the following text:

"Due the authors' interest in investigating psychological interventions targeting dancers at professional, pre-professional and recreational levels, clinical populations (e.g., individuals that engage in dance for health, rehabilitation, or therapy approaches) will be excluded from this review. "

Eligibility criteria – intervention & outcome
40. In intervention, you state that interventions that are dance technique/motor skill focused will be excluded. In outcomes you state that relevant studies will seek to improve dancer's physical health. Is this because some mental skills/techniques could have physical outcomes? If yes, please make it clear in intervention or outcome.

From a preliminary scoping of the literature, a few MST interventions target dancers' mental health regarding recovering from injuries. Therefore, we wanted to incorporate this aspect into the review as an indicator of health. To address this concern and to also address comments from reviewer 1, we have amended the outcome section to:

Please see our response to Comment #10 from Reviewer 1 .

Information sources
41. Please update the end date with the acceptance of this protocol.

Amended in the revised manuscript.

Search strategy
42. What search strategy will you follow in Google Scholar?

This (and the missing Embase search string) has been included in table 1 of the amended manuscript.

Selection process
43. Please define PRISMA in the Data management paragraph and use only abbreviation thereafter.

Amended in the revised manuscript.

Data collection process
44. Second line, the reference does not seem to be well formatted.

Amended in the revised manuscript.

45. Table 2: Will you include n?
Thank you. N has been added to the table.
